# Premalignant alteration assessment in liver-like tissue derived from embryonic stem cells by aristolochic acid I exposure

**DOI:** 10.18632/oncotarget.12424

**Published:** 2016-10-04

**Authors:** Tong Li, Ke Jin, Dan-yan Zhu, Lu Li, Zheng-rong Mao, Bo-wen Wu, Yi-fan Wang, Zong-fu Pan, Lan-juan Li, Chun-sheng Xiang, Kun-kai Su, Yi-jia Lou

**Affiliations:** ^1^ Institute of Pharmacology and Toxicology, College of Pharmaceutical Sciences, Zhejiang University, Hangzhou 310058, China; ^2^ Laboratory of Pathology and Pathologic Physiology, Department of Basic Medicine, College of Medicine, Zhejiang University, Hangzhou 310058, China; ^3^ State Key Laboratory for Diagnosis and Treatment of Infectious Diseases, The 1st Affiliated Hospital, College of Medicine, Zhejiang University, Hangzhou 310003, China; ^4^ Collaborative Innovation Center for Diagnosis and Treatment of Infectious Diseases, The 1st Affiliated Hospital, College of Medicine, Zhejiang University, Hangzhou 310003, China

**Keywords:** remalignant assessment, stem cell-derived liver-like tissue, paracrine IL-6, c-Myc/Lin28B, aristolochic acid I

## Abstract

The *in vitro* predictive evaluation of chemical carcinogenicity based on hepatic premalignance has so far not been established. Here, we report a novel approach to investigate the premalignant events triggered by human carcinogen aristolochic acid I (AAI) in the liver-like tissue derived from mouse embryonic stem cells. By AAI exposure, the liver-like tissue exhibited the paracrine interleukin-6 phenotypic characteristics. Hepatocytes expressed STAT3/p-STAT3, c-Myc and Lin28B in parallel. Some of them displayed the dedifferentiation characteristics, such as full of α-fetoprotein granules, increase in size, and nucleocytoplasmic shuttle of Oct4. When these cells were injected into mice, the xenografts mostly displayed the uniform area of hepatic-like tissue with malignant nuclei. The hepatic malignant markers, α-fetoprotein, cytokeratin 7 and cytokeratin 19, were co-expressed in albumin-positive areas, respectively. In conclusion, we established an approach to predict the hepatic premalignance triggered by carcinogen AAI. This premalignant assay system might aid to evaluate the effects of potential carcinogens in liver, and probably to screen the protecting against hepatocarcinogenic efficacy of pharmaceuticals *in vitro*.

## INTRODUCTION

Hepatocellular carcinoma (HCC) is one of the most lethal malignancies [1, 2]. Premalignant alterations are believed to take a pivotal role in the pathological processes of HCC [2–4]. To date, the evaluation for chemical carcinogenicity is based on the 2-year rodent bioassay [5]. In addition to its expensive cost, labor and time consumption, such a bioassay hardly demonstrates the premalignant events. Therefore, seeking a reliable *in vitro* mammal model would be valuable for looking into the early pathological alterations of chemical-induced hepatic carcinogenesis.

Acquired autocrine interleukin (IL)-6 signaling stimulates HCC progenitor cells or fully malignant HCC, but paracrine IL-6 by inflammatory cells directly contributes to hepatocarcinogenic initiation [6]. Mouse embryonic stem (ES) cell-derived liver-like tissue could be an option for an *in vitro* paracrine IL-6-producing model. The hepatocytes generated from ES cells in culture exhibit many phenotypic characteristics of primary adult hepatocytes. The tissue structurally consists of a blood/sinusoid vascular-like network and hepatocyte layers, and shows higher levels of hepatic function [7]. Our and others studies revealed that the liver-like tissue possesses the 5-lipoxygenase pathway, metabolic functions of cytochrome P_450_ family and phase II metabolic enzymes at the terminal differentiation stage [8–11]. Therefore it has been becoming an *in vitro* hepatic pathophysiological and drug metabolism model. In the present study, we further presume that the sinusoid-like structure probably also contains Kupffer-like cells and potentially produces cytokines, including IL-6. Chemicals exposure might trigger the inflammatory process via IL-6 paracrine way in the liver-like tissue. ES cell-derived hepatocytes have been used to evaluate the carcinogenic hazard of chemicals using Affymetrix gene microarray measurements based on applying an ANOVA model [5]. But pure hepatocytes do not possess the tissue function, and hardly show the ascendancy described above.

C-Myc oncoprotein overexpression can induce HCC with a high frequency in mice [12]. Activation of c-Myc oncogenic transcription factor (Myc) induces oncofetal RNA-binding protein Lin28B expression in multiple human and mouse tumor models [13]. Obviously, in the case of IL-6 paracrine and c-Myc/Lin28B signal activation in the derived liver-like tissue, the upstream or downstream molecular events will form crosstalks for amplification and feedbacks of hepatic premalignance. α-fetoprotein (AFP) is one of the well accepted malignant biomarkers in mature liver tissue. Thus, c-Myc and Lin28B coupled with AFP expression could be closely correlative with the outcomes of premalignant alterations in the mature liver-like tissue. Human carcinogen aristolochic acid I (AAI) existing in plant drugs from *Aristolochia* species is an environmental human carcinogen associated with urothelial cancer. Short-term AAI exposure displays potential hepatocarcinogenesis *in vivo* and *in vitro* [14–16]. Recently, our study demonstrated that hepatic premalignant alterations appeared in canines after a 10-day AAI oral administration, featured by c-Myc oncoprotein and Lin28B overexpressions and cancer progenitor-like cell formation [17].

In the present study, we demonstrated the IL-6 paracrine and c-Myc/Lin28B/AFP expression in the mouse ES cell-derived liver-like tissue by AAI exposure. We also confirmed that the subsequent xenografts derived from AAI-exposed cultivation exhibited the typical premalignant markers within albumin (ALB) staining areas in mice. The results underscore the similarities of liver-like tissue derived from ES cells to the liver tissues of mice or canines *in vivo* [14, 17, 18]. This novel *in vitro* mammal test system, therefore, could be beneficial in providing an optimal platform to explore the hepatic premalignant transformation triggered by chemicals and screen the protecting against carcinogenic efficacy of pharmaceuticals.

## RESULTS

### Evaluation criteria by suitable concentration of AAI exposure

**Figure 1:**
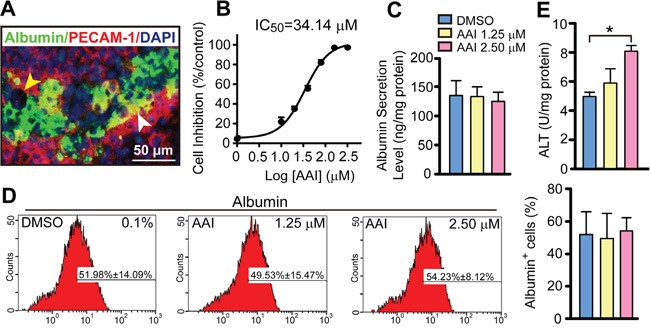
Quantitative evaluation of biological features in the liver-like tissue derived from ES cells with or without AAI exposure **A.** The morphological mosaic feature of the liver-like tissue at the differentiation endpoint using immunofluorescence of ALB and PECAM-1 without AAI (white arrowhead: sinusoid vascular-like network, yellow arrowhead: bile-duct-like structure). **B.** The cytotoxic effect of AAI on ES cells (with IC_50_) measured after a 7-day incubation. **C.** ALB level in supernatants of the liver-like tissue culture medium treated with or without AAI. **D.** Quantification of hepatocytes with ALB marker in the liver-like tissue using flow cytometry assay. **E.** ALT level in supernatants of the liver-like tissue culture medium treated with or without AAI. Error bars represent the mean value ±S.D. *P<0.05 *vs* DMSO. For each analysis *n*=3.

At the endpoint after the onset of differentiation, the embryoid body (EB) outgrowths exhibited a blood/sinusoid vascular-like network and hepatocyte layers, i.e. the typical liver-like tissue morphology (Figure 1A). The cytotoxicity of AAI on ES cells was measured, and the 50% inhibition concentration (IC_50_) of AAI was 34.14 μM at a 7-day incubation (Figure 1B). To distinguish whether the differentiation ability was decreased by AAI exposure, AAI was used with indicating final concentrations. The liver-like tissue exhibited structural disorder in 5.00 μM or 10.00 μM AAI-containing medium (Supplementary Figure S1). When incubated with 1.25 μM or 2.50 μM AAI, EBs could differentiate in culture into liver-like tissue. ALB synthesis and secretion functions, the unique features of mature hepatocytes, were stable by flow cytometry and ELISA assessments (Figure 1C, 1D). But alanine transaminase (ALT) level in supernatants was increased by 2.50 μM AAI treatment (*P*<0.05, Figure 1E), indicating the plasma membranes of hepatocytes were slightly injured. Here, we used 2.50 μM as a suitable concentration for the premalignant biomarker evaluation.

### AAI treatment causes premalignant alterations in mature liver-like tissue

**Figure 2:**
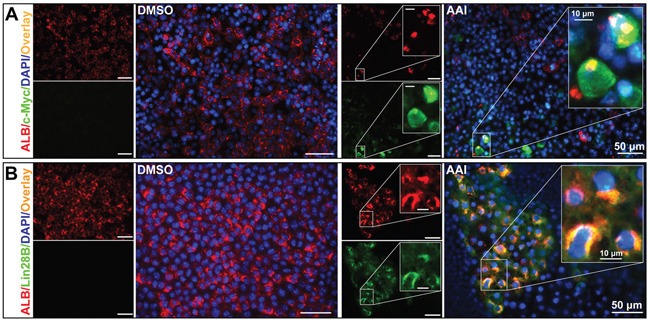
Premalignant alteration events triggered by AAI exposure in the liver-like tissue at the differentiation endpoint **A.** ALB residues (red) appeared in c-Myc-positive cells (green). **B.** Lin28B (green) co-expressed with ALB (red) in cytoplasm of hepatocytes.

Immunofluorescence staining revealed that several c-Myc-positive cells appeared in the ALB-positive areas in response to AAI treatment. More importantly, a few ALB residues existed in the cytoplasm of c-Myc-positive cells (Figure 2A). The ALB and c-Myc co-expression ratio was approximately 10.0% (Table 1), indicating that it was hepatocytes that possessed the oncogenic susceptibility to AAI exposure. Lin28B also co-expressed with ALB in cytoplasm (Figure 2B), and the double-staining ratio reached by 12.5% (Table 1). This phenomenon was consistent with that in canine livers after AAI oral administration [17]. Considering that a c-Myc/Lin28B oncogenic circuit was necessary and sufficient to transform cells [19], here, both c-Myc and Lin28B could be considered as the markers of carcinogenesis by AAI exposure in the liver-like tissue.

**Table 1:** A schedule of key hallmarks of premalignant transformation with immunofluorescence analysis in ES cellderived liver-like tissue by AAI exposure

Co-expression of markers	Batch of cultures		By AAI exposure		Positive ratio (%)
	DMSO	AAI	Total visual field numbers	Total positive visual field numbers	
ALB+c-Myc	3	3	30	3	10.0
ALB+Lin28B	5	5	40	5	12.5
ALB+IL-6	3	3	39	36	92.3
ALB+AFP	3	3	53	6	11.3

12 wells per batch for each marker combination.

### Dedifferentiation of hepatocytes by AAI exposure in mature liver-like tissue

**Figure 3:**
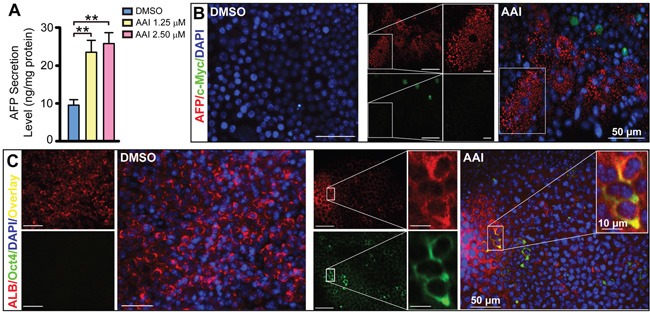
The dedifferentiation of hepatocytes by AAI exposure in mature liver-like tissue at the terminal differentiation stage **A.** AFP level in supernatants of the liver-like tissue culture medium treated with or without AAI. Immunofluorescence staining showed when treated with AAI: **B.** the immature marker AFP-positive cells (red) appeared in c-Myc-positive cells (green) area and increased in size; **C.** Oct4 (green) co-expressed with ALB (red) in cytoplasm of hepatocytes. Error bars represent the mean value ±S.D. **P<0.01 *vs* DMSO. For each analysis *n*=3.

ELISA assay demonstrated that AFP levels in supernatants were increased by 2.4-fold or 2.6-fold in response to 1.25 μM or 2.50 μM AAI treatment (*P*<0.01, Figure 3A). Interestingly, some cells were rich in AFP granules in cytoplasm, increased in size, and existed with c-Myc-positive cells (Figure 3B), which was most likely hepatocytes in premalignant transformation [20, 21]. Furthermore, transcription factor octamer-binding transcription factor (Oct)-4 co-expressed with ALB in cytoplasm (Figure 3C). Oct4 participates in cell reprogramming rather than self-renewal based on its nucleocytoplasmic shuttling property [22]. Our results implied that Oct4 in cytoplasm contributed to the dedifferentiation of mature hepatocytes.

### Paracrine IL-6 signaling activation in liver-like tissue by AAI exposure

**Figure 4:**
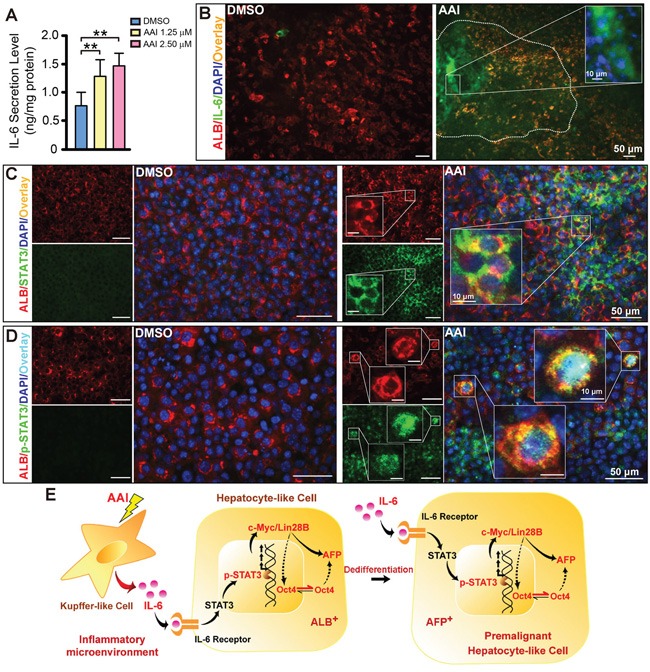
Inflammatory cytokine IL-6 signaling activation in the ES cell-derived liver-like tissue treated with AAI **A.** IL-6 level in supernatants of the liver-like tissue culture medium treated with or without AAI. Immunofluorescence staining showed: **B.** widespread IL-6 (green) appeared in the hepatocyte area (red) with the enriched abundance in the sinusoid-like structure; **C.** up-regulated STAT3 (green) existed in cytoplasm of hepatocytes (red); **D.** robustly up-regulated p-STAT3 (green) appeared in nuclei of hepatocytes (red). **E.** The schematic signaling mechanism of the AAI-induced premalignant alterations in the ES cell-derived liver-like tissue. Error bars represent the mean value ±S.D. **P<0.01 *vs* DMSO. For each analysis *n*=3.

Using ELISA assay, we found IL-6 level in supernatants double increased in response to 2.50 μM AAI (*P*<0.01), and exhibited a concentration-dependent manner by AAI exposure (Figure 4A). The widespread IL-6 also appeared in the liver-like tissue (Figure 4B). Totally, 92% of visual fields displayed IL-6 expression in 48-well plates from several preparations (Table 1). We therefore investigated whether enhanced paracrine IL-6 contributed to c-Myc/Lin28B expression in the liver-like tissue. We found that its downstream event STAT3 was up-regulated in cytoplasm of hepatocytes (Figure 4C), and that p-STAT3 overexpressed and appeared in a few nuclei of hepatocytes in parallel (Figure 4D). The phenomena implied that IL-6 signaling was activated in the liver-like tissue by AAI exposure. Unexpectedly, NF-κB (p65) was only slightly up-regulated and in major co-expressed with ALB in cytoplasm of hepatocytes (Supplementary Figure S2). The schematic depiction represents the *in vitro* events of hepatocarcinogenic pathways by AAI exposure (Figure 4E).

### Hepatocarcinoma within xenografts with premalignant phenotypic cells

**Figure 5:**
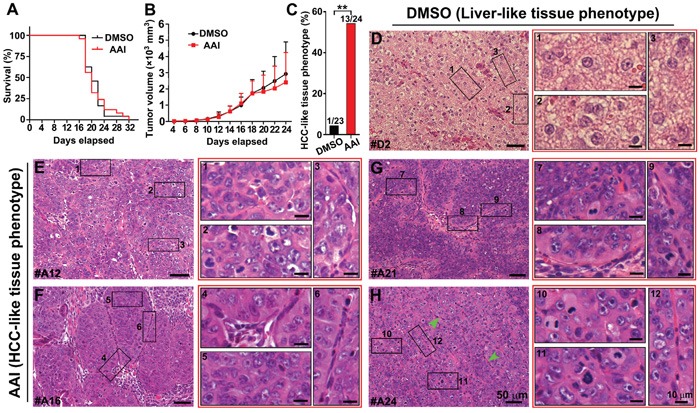
*In vivo* validation of tumorigenicity of premalignant cells in the liver-like tissue Cells from both DMSO- and AAI-incubated EBs were injected into the back of mice at the indicated time. **A.** The survival curve of NOD/SCID mice bearing xenografts. **B.** The growth curve of xenograft volume. **C.** The ratio of the HCC-like tissue phenotype of the xenografts in the DMSO and AAI group (*n*=23⊼24/group). **D.** The characteristic liver-like tissue phenotype of the xenografts in the DMSO group. **E.** to **H.** Remarkable uniform areas of hepatic-like cells with malignant nuclei appeared in the xenografts of AAI-exposed EBs. Error bars represent the mean value ±S.D. **P<0.01 *vs* DMSO.

Cells from both DMSO- and AAI-incubated outgrowth of EBs were injected into the back of mice. The ratio of survived mice with different xenografts in both groups was very similar. Moribund mice with xenografts firstly appeared on day 16 (Figure 5A). The xenografts displayed the similar size in both groups at the indicated time (Figure 5B). The ratio of xenografts with HCC-like phenotypic sections robustly increased, and reached 54% (13/24) in AAI xenografts compared with DMSO xenografts (1/23) (*P*<0.01, Figure 5C). Using H&E staining, the liver-like tissue was displayed in xenografts of DMSO control (Figure 5D). By contrast, uniform area of hepatic-like cells with malignant nuclei appeared in the xenografts growing from AAI-exposed EBs. The cells exhibited bigger and darkly-stained nuclei, arranging in disorder, pseudolobule or microvascular invasion (Figure 5E to 5H). These data suggested that hepatocarcinoma formed in xenografts injected with premalignant phenotypic cells. We also found the teratoma formed in both xenografts. Compared with DMSO control, the teratoma in AAI xenografts showed the much smaller size (Supplementary Figure S3), implying that the cells in HCC-like phenotypic xenografts probably possess the short cell cycle and the rapid proliferation features [23].

### AFP, K7 and K19 expression in xenografts with malignant phenotypic cells

**Figure 6:**
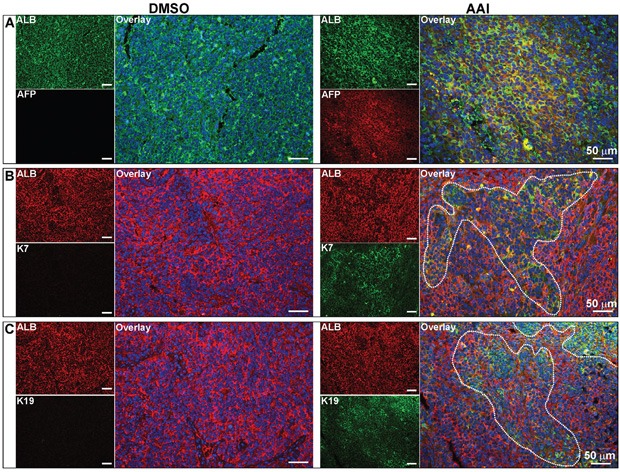
Expression of hepatic malignant markers in the xenografts Immunofluorescence staining showed the co-expression of AFP **A.**, K7 **B.** and K19 **C.** with ALB in xenografts with malignant phenotypic cells. For DMSO and AAI group, *n*=23 and 24 respectively.

Both cytokeratin 7 (K7) and K19, the biliary/hepatic progenitor cell markers, were expressed in a subset of HCC with poor prognosis [24, 25]. Double-immunofluorescence staining revealed that xenografts with malignant phenotypic cells co-expressed ALB with AFP, K7 and K19, respectively (Figure 6A to 6C). The data confirmed that xenografts with HCC-like phenotype could develop into HCC with poor prognosis.

### General information on hepatic premalignance *in vitro* and two-segment workflow

Employing the *in vitro* test system, a set of predictive characteristics based on the c-Myc/Lin28B/AFP expression has been evaluated in the present study. Here we summarize the data observed in the liver-like tissue incubated with AAI. The major positive markers and their percentage have been concluded in Table 1. The individual expression and the occurrence rate of the tested biomarkers at different segments have also been listed in Supplementary Table 1.

**Figure 7:**
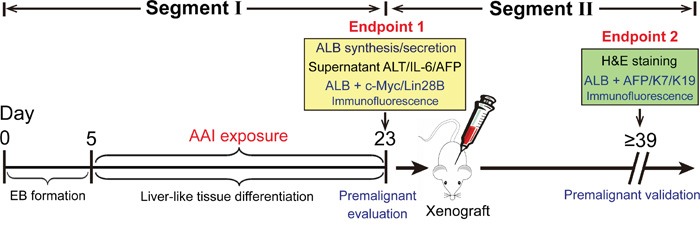
Schematic depiction of procedures for assessment and validation of hepatic premalignance in liver-like tissue derived from mouse ES cells

By AAI exposure, the mouse ES cell-derived liver-like tissue generation and the premalignant event evaluation were described in the workflow (Figure 7, Segment I). If the suggested premalignant landmarks appeared in the liver-like tissue, the *in vivo* validation should be further considered in mice (Figure 7, Segment II).

## DISCUSSION

To date, little is known about the assessment for initial events of chemical hepatocarcinogenicity *in vitro*. Therefore, seeking a suitable model is quite necessary and urgent for either pathological or pharmacological research purposes. Short-term AAI exposure displays potential hepatocarcinogenesis *in vivo* and *in vitro* [14–16]. Our previous study demonstrates that hepatic premalignant alterations appear in canines after a 10-day AAI oral administration. It is featured by c-Myc oncoprotein and Lin28B overexpressions and cancer progenitor-like cell formation in the liver [17]. Although the major signaling pathways have been illustrated in that case, the premalignant node is still not applied *in vitro*. Therefore, here we further employ an *in vitro* approach to confirm AAI-induced hepatocarcinogenic susceptibility.

The liver-like tissue displays significantly higher ALB expression than hepatic cell lines and primary cultures of murine adult hepatocytes [7]. The present study shows 50% ALB-positive cells in the tissue. The tissue stably released ALB to supernatants, which therefore can be quantitatively employed to mimic the serum ALB level measurement *in vivo*. This *in vitro* model offers the structure for paracrine IL-6, and probably other cytokines by potential hepatocarcinogen exposure. Present result indicates that IL-6 in supernatants is significantly increased in an AAI concentration-dependent manner. Although AAI exhibits higher cytotoxicity in liver cells than that in kidney cells [26], it does not reduce the differentiation ratio of EB cultivations in very low concentration of AAI (less than 8% IC_50_) exposure. Taking the *in vivo* signaling activation as a clue [17], the *in vitro* model reflects three related aspects, that is, paracrine IL-6 related inflammatory activation, premalignant transformation, and hepatocyte dedifferentiation. AAI is served as the tool compound, and the expected biomarkers can be identified at the differentiation endpoint of liver-like tissue. ALB, IL-6 or AFP in supernatants can be firstly selected for evaluating the ALB synthesis and secretion function, as well as inflammatory activation or premalignant transformation in hepatocytes, respectively. The assessment is easy to handle and hopeful to develop into a routine method.

The present study demonstrates AFP-positive cells appear together with c-Myc-positive cells, with granules in cytoplasm and increase in size. It has been reported that most well-differentiated HCCs in the early stages are detected as small nodules with normal levels of AFP [27]. Subsequently, these malignant cells increase in size and become moderately or poorly differentiated cancerous tissues producing AFP [20]. In the present study, we also find Oct4 nucleocytoplasmic shuttling when incubated with AAI. Oct4 is imported into the nucleus where it functions as a transcription factor; however, it possesses the spatiotemporal dynamic behavior. In the case of Oct4 localized in the cytoplasm, it involves the chromatin remodeling and/or epigenetic changes of cells, and contributes to somatic cell reprogramming and determination of cell fate [22].

Paracrine IL-6 released by Kupffer cells is a pro-inflammatory cytokine initiating inflammatory response in liver. STAT3 pathway can be triggered via binding IL-6 receptor. Once recruited, it is phosphorylated on specific tyrosine residues, thus allowing their dimerization and translocation to the nucleus [28]. The present study demonstrates both STAT3 and p-STAT3 are up-regulated in hepatocytes of the liver-like tissue after AAI treatment. Importantly, several hepatocytes display p-STAT3 translocation to the nuclei. NF-κB (p65) is only slightly up-regulated in cytoplasm of hepatocytes, suggesting it might not be activated in the liver-like tissue by AAI exposure. Paracrine IL-6 itself probably directly triggers p-STAT3 and Lin28B expression in this case. Malignant phenotype will be advanced when shifting from paracrine IL-6 to c-Myc/Lin28B via p-STAT3 activation *in vitro*. It means IL-6 may trigger epigenetic changes to increase the transcriptional expression of oncogenes and activate oncogenic signaling pathways in the *in vitro* system. Thus, IL-6-induced Lin28B expression seems a complementary manner via Oct4-associated role in the somatic cell reprogramming *in vitro*, resulting in hepatocyte dedifferentiation. The data are strongly correlated with those *in vivo* experiments in receiving AAI, and potentially reflect the possible mechanisms of pathological processes in the chemical-induced hepatocarcinogenesis.

In addition, compared with DMSO control, hepatocarcinoma ratio increases in xenografts injected with premalignant phenotypic cells by AAI exposure. The reason is probably that the cells in HCC-like phenotypic xenografts possess the short cell cycle and the rapid proliferation features [23]. The xenografts with malignant phenotypic cells also co-express ALB with the biliary/hepatic progenitor cell markers AFP, K7 and K19, and might develop into HCC with poor prognosis [24, 25]. These *in vivo* outcomes confirm that the results from *in vitro* system are credible.

In summary, our results demonstrate the characteristics of hepatic premalignant alterations triggered by AAI in the mouse ES cell-derived liver-like tissue. The method basically contains a two-segment workflow for evaluation and validation. These findings not only mimic the pathological events of initiating process of premalignance *in vivo*, but also reveal fundamental criteria for the established hepatic microenvironment *in vitro*. This approach could provide a set of correlate and evaluative biomarkers in different research fields. Therefore the platform allows evaluating either hepatic pathological processes or inhibiting effects of drugs on the different segments of hepatocarcinogenicity.

## MATERIALS AND METHODS

### ES cell viability assay by AAI exposure

The permanent mouse ES cell line D3 was obtained from the American Type Culture Collection (CRL-1934, Manassas, VA, USA) and maintained as previous described [29]. AAI powder (purity >98%, HPLC, Delta, China) was dissolved in DMSO to obtain the stock solution (80 mM), which was stored at -20°C. For *in vitro* cell treatment, ES cells were planted into 96-well plate and incubated with the media containing the gradiently-diluted concentrations of AAI from the stock solution. After treatment for 7 days, cell viability was determined by MTT (Amresco, LLC, MA, USA) assay. Assays were run in three independent experiments, and each condition was repeated in six technical replicates. The IC_50_ was calculated [30].

### ES cell differentiation and AAI exposure

Differentiation of ES cells was initiated by formation of EBs in hanging drop culture as previously described [10]. Briefly, drops of 30 μl containing about 600 ES cells were placed on the lids of Petri dishes, and cultured in hanging drops for 5 days. After EB formation, 15 EBs or 2 EBs were plated into 6-well or 48-well plates, respectively. D 5 was referred to the day of EB plating culture, and EBs were incubated with 1.25 μM or 2.50 μM AAI (final concentrations) from d 5 to d 23. EBs in the control group were incubated with the appropriate amount of vehicle (0.1% DMSO) in parallel, which did not decrease the cell viability.

### Flow cytometric analysis

The flow cytometric analysis was conducted as previously described [11]. Generally, the EBs in 6-well plates treated with AAI or DMSO at d 23 were harvested and digested with Accutase (Millipore, Billerica, MA, USA) to generate single cell suspension. Then cells were fixed with 4% (wt/vol) paraformaldehyde (PFA) (pH 7.4) at room temperature for 60 min. After being rinsed three times with D-Hank's solution (pH 6.8), cells were blocked with 3% bovine serum albumin (BSA) supplemented with 0.05% Triton X-100 for 60 min. Then specimens were incubated with appropriate dilution of the rabbit anti-mouse primary antibody to ALB (ab19196, Abcam, Cambridge, MA, USA) at 4°C overnight and the diluent was incubated with cells as the negative control. On the next day, specimens were incubated with Dylight 488-conjugated goat anti-rabbit secondary antibody for 20 min on the ice and finally resuspended in 500 μl D-Hank's solution. The ratio of hepatocyte differentiation was measured with a flow cytometer (FACScan, Becton Dickinson, Heidelberg, Germany).

### Immunofluorescent assay

The immunofluorescent assay was performed as previously described [10]. In brief, for immunofluorescence of cell culture, EBs treated with AAI or DMSO grown in 48-well plates at d 23 were washed three times with D-Hank's solution and fixed with PFA at room temperature for 20 min. After being rinsed twice with D-Hank's solution, cells were incubated with 3% BSA supplemented with 0.05% Triton X-100 for 60 min. Then specimens were incubated at 4°C overnight with the following primary antibodies: antibodies against ALB (ab19196, ab19194), platelet endothelial cell adhesion molecule 1 (PECAM-1, ab24590), IL-6 (ab6672), c-Myc (ab32072), Oct4 (ab59545), K7 (ab181598) and K19 (ab52625) were obtained from Abcam; antibodies against STAT3 (*12640), p-STAT3 (*9145), NF-κB (p65, *4764) and NF-κB (p50, *3035) were purchased from Cell Signaling Technology (Danvers, MA, USA); antibodies against AFP (sc-8108) was purchased from Santa Cruz Biotechnology (CA, USA); antibody against Lin28B (HPA036630) was purchased from Sigma Aldrich (Atlas antibodies, St. Louis, MO, USA). On the next day, specimens were incubated with the corresponding secondary antibodies (Dylight 488 or 549-conjugated goat anti-mouse or goat anti-rabbit secondary antibodies from MULTISCIENCES and Cy3 or FITC-conjugated donkey anti-goat or donkey anti-rabbit secondary antibodies from Abcam) for 2 h at room temperature. DAPI (Sigma) was stained to represent nuclei of cells. Specimens were observed using a fluorescence microscope equipped for fluorescence analysis (Leica DMIL, Wetzlar, German). Only ALB-positive areas were considered as the visual fields, and biomarkers were further evaluated in the areas.

For immunofluorescence of xenograft paraffin sections, slices of samples were firstly heated in a 60°C drying oven for 30 min and deparaffined in xylene, and rehydrated through a series of decreasing concentrations of ethanol. The following procedures were performed as described above. At last, the slices were mounted with Anti-fade Fluorescence Mounting Medium (Beyotime Biotechnology, China) and observed using the fluorescence microscope described above.

### ALB, ALT, AFP and IL-6 secretion determination

The supernatant samples of cell cultures treated with AAI or DMSO in 6-well plates were collected at the d 23 (media finally changed two days before the harvest). The secretion of ALB, AFP and IL-6 was analyzed using Mouse Albumin ELISA Kit (ab108792, Abcam), Mouse α-Fetoprotein/AFP Quantikine ELISA Kit (MAFP00, R&D Systems, USA) and Mouse IL-6 ELISA Kit (ab100712, Abcam) respectively, and the experiment was performed as the manufacturer's instructions. The ALT levels in the supernatants were measured using an automatic biochemical analyzer (Olympus AU5431, Olympus Corporation, Tokyo, Japan). Assays were run in three independent experiments, and each condition was repeated in three technical replicates. Released ALB, ALT, AFP and IL-6 were normalized to total protein content.

### Ethics statement

All experimental procedures involving animals were approved by Institutional Animal Care and Use Committee of Zhejiang University (Ethics Code: ZJU201411-1-01-101) and performed in accordance with the institutional ethical guidelines for animals.

### Xenograft model for tumorigenicity

Fifty-two six-week old male NOD/SCID mice were purchased from National Resource Center for Rodent Laboratory Animal (Shanghai branch, China) and maintained in a specific pathogen-free environment. The mice were randomized to DMSO and AAI groups. The differentiation cultures of EBs treated with AAI or DMSO for 18 days were harvested and digested with Accutase and then filtered through a 200 μm sieve to generate single cell suspension. Approximately 4×10^6^ cells in 200 μl culture medium were injected subcutaneously into the back of mice. After four days from initiation of injection, mice were weighed and the tumor width (W) and length (L) were measured every two days. Tumor volume was estimated according to the standard formula L×W^2^/2 [31]. After two weeks, when mice would be dying or their tumors displayed ulcer, they would be euthanized. Tumors were excised immediately after sacrifice. Part of the tumors was fixed in 4% PFA for H&E and immunofluorescent analyses.

### H&E staining assay

The H&E staining was carried out as previously described [32]. Generally, tumor sections from DMSO and AAI groups were embedded with paraffin, and then cut into 5 μm-thick slices. Then tissue samples were routinely deparaffined and rehydrated, and finally stained with hematoxylin and eosin to evaluate tumor structure.

### Statistical analysis

All experiments were reproduced three times and all data were analysed using GraphPad Prism 5 (GraphPad software, San Diego, CA, USA) and expressed by mean ± S.D. A two-sided Student's *t*-test was used to determine the statistical significance of differences between experimental and control groups. *P<0.05, **P<0.01 was considered statistically significant.

## SUPPLEMENTARY FIGURES AND TABLES


