# Supplementary material for: Premalignant alteration assessment in liver-like tissue derived from embryonic stem cells by aristolochic acid I exposure
Source: Oncotarget. 2016 Oct 4;7(48):78872–82. doi: 10.18632/oncotarget.12424 (PMC5346684; doi:10.18632/oncotarget.12424)
Supplement: Supplementary file 1 [file oncotarget-07-78872-s001.pdf]

Premalignant alteration assessment in liver-like tissue derived from embryonic stem cells by aristolochic acid I exposure

SUPPLEMENTARY FIGURES AND TABLE

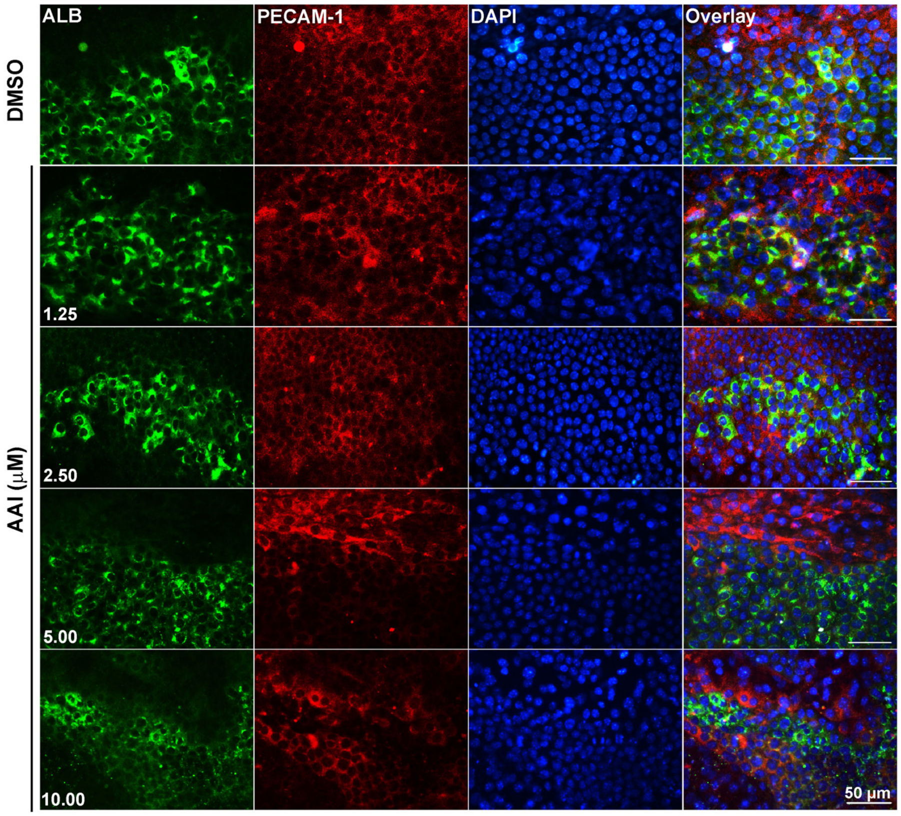

Supplementary Figure S1: Effects of different AAI concentrations on liver-like tissue structure in mouse ES cell-derived *in vitro* liver-like tissue system. When AAI concentration increased up to 5.00 μM, the sinusoid vascular-like network was disrupted.

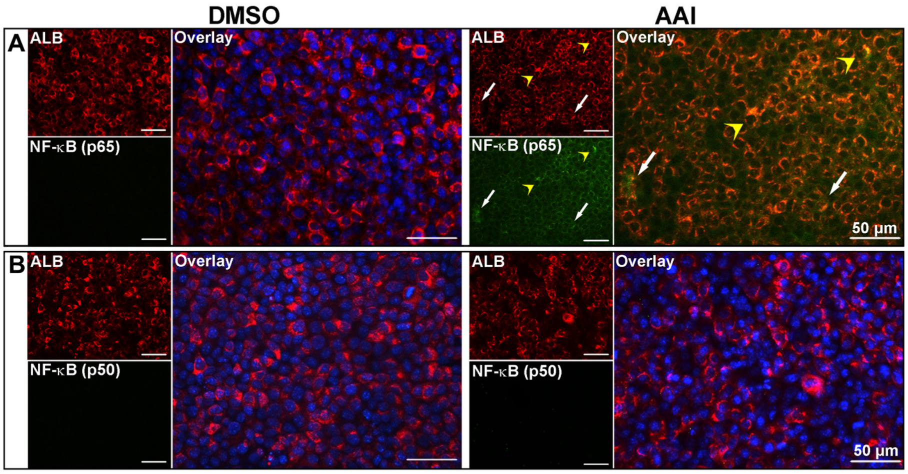

Supplementary Figure S2: Expression of NF-κB (p65, p50) in the mouse ES cell-derived liver-like tissue with or without AAI at the differentiation endpoint. A. NF-κB (p65) was slightly up-regulated (green) and in major co-expressed with ALB (red) in cytoplasm (arrow head) or a few in nuclei (arrow) of hepatocytes by AAI exposure. **B.** NF-κB (p50) could almost not be detected in the liver-like tissue by immunofluorescence.

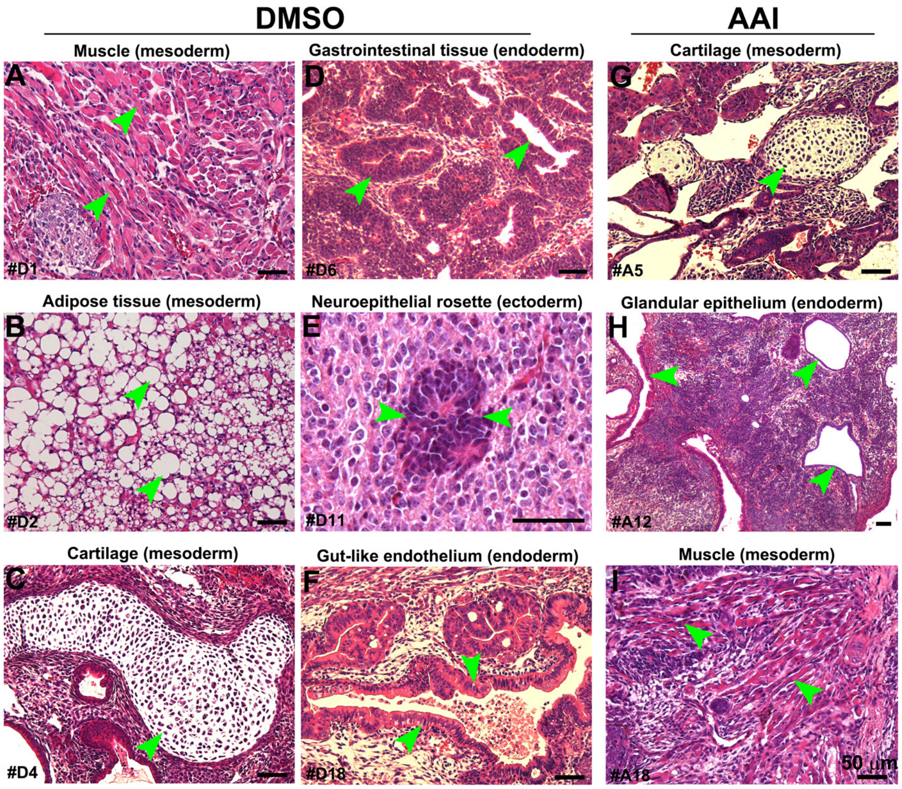

Supplementary Figure S3: The H&E staining of the teratoma phenotypes in the xenografts of DMSO and AAI group. DMSO: **A.** muscle (mesoderm), **B.** adipose tissue (mesoderm), **C.** cartilage (mesoderm), **D.** gastrointestinal tissue (endoderm), **E.** neuroepithelial rosette (ectoderm), **F.** gut-like endothelium (endoderm). AAI: **G.** cartilage (mesoderm), **H.** glandular epithelium (endoderm), **I.** muscle (mesoderm).

Supplementary Table S1: Expressions of predictive markers in the liver-like tissue derived from mouse ES cells by AAI exposure

Hallmarks (proteins)	Mature hepatocytes	Inflammatory activation	Premalignant transformation
ALB	+++	-	-
IL-6	-	+++	+
STAT3	-	+	-
p-STAT3	-	++	-
NF-κB (p65)	-	+ (cytoplasm)	- (nuclei)
c-Myc	-	-	++
Lin28B	-	-	++
AFP	-	-	+++
Oct4	-	-	+ (cytoplasm)
